# Improving the Detection Limit in a Capillary Raman System for *In Situ* Gas Analysis by Means of Fluorescence Reduction

**DOI:** 10.3390/s150923110

**Published:** 2015-09-11

**Authors:** Simone Rupp, Andreas Off, Hendrik Seitz-Moskaliuk, Timothy M. James, Helmut H. Telle

**Affiliations:** 1 Institute for Technical Physics, Tritium Laboratory Karlsruhe, Karlsruhe Institute of Technology, 76021 Karlsruhe, Germany; E-Mails: Andreas.Off@kit.edu (A.O.); Hendrik.Seitz-Moskaliuk@kit.edu (H.S.M.); tjames@laserquantum.com (T.M.J.); 2 Instituto Pluridisciplinar, Paseo Juan XXIII-1, Universidad Complutense, 28040 Madrid, Spain; E-Mail: helmut@telle-online.eu

**Keywords:** Raman spectroscopy, capillary, gas analysis, process control, real-time monitoring, instrument development, fluorescence reduction

## Abstract

Raman spectroscopy for low-pressure or trace gas analysis is rather challenging, in particular in process control applications requiring trace detection and real-time response; in general, enhancement techniques are required. One possible enhancement approach which enjoys increasing popularity makes use of an internally-reflective capillary as the gas cell. However, in the majority of cases, such capillary systems were often limited in their achievable sensitivity by a significant fluorescence background, which is generated as a consequence of interactions between the laser light and optical glass components in the setup. In order to understand and counteract these problems we have investigated a range of fluorescence-reducing measures, including the rearrangement of optical elements, and the replacement of glass components—including the capillary itself—by metal alternatives. These studies now have led to a capillary setup in which fluorescence is practically eliminated and substantial signal enhancement over standard Raman setups is achieved. With this improved (prototype) setup, detection limits of well below 1 mbar could be obtained in sub-second acquisition times, demonstrating the potential of capillary Raman spectroscopy for real-time, *in situ* gas sensing and process control applications, down to trace level concentrations.

## 1. Introduction

Raman spectroscopy is widely used as an analytical tool to identify and also quantify molecular constituents in liquid and solid samples. With the advancement in laser and photon detector technology, it also has become increasingly popular for gas analysis applications. A major advantage is the possibility for *in situ* gas monitoring, which is of significance in a range of diagnostic and process control applications (see [1,2]). Unlike infrared absorption spectroscopy, Raman spectroscopy is sensitive to important homonuclear molecular gases, such as hydrogen, nitrogen and oxygen [3]. Furthermore, it provides excellent spectral “fingerprints” of molecular gas species, which allow for quantitative compositional analysis; in many cases, even the distinction of isotopologues of individual species is achievable [4].

However, since Raman scattering cross-sections are very small, this poses a severe challenge in low pressure (hence low total particle density) applications. In particular, for real-time process monitoring or the detection of trace gases at partial pressures in the mbar or sub-mbar range, measurement systems with extreme sensitivity are required. While a range of Raman enhancement techniques exist, such as cavity-enhanced, stimulated or surface-enhanced Raman scattering [5,6,7], most require that stringent tolerances be met by the system (e.g., concerning system stabilization), or they may be restricted to detect just a few, selected molecular species.

A simple, robust approach to significantly enhance the Raman signal utilizes a hollow tube/capillary with a reflective inner surface as the gas cell, through which the laser beam passes longitudinally; this was first suggested in the mid-1990s [8]. In the proposed generic setup, the laser light is confined in the capillary and interacts with the gas molecules over the entire length of the capillary, greatly increasing the interaction region compared to free-space solutions. The Raman light is collected in a backward configuration, with the reflective capillary tube guaranteeing as well that the scattered light is collected over the full length.

A few research groups, including ourselves, have built on the principle configuration proposed by Carlsen. For example, Pearman *et al.* reported up to 20-fold signal enhancements for a fiber-optic probe; they used an internally silver-coated glass capillary of 2 mm inner diameter as the gas cell [9]. For their direct-focusing metal-lined glass capillary setup, Buric *et al.* achieved a 1σ detection limit of 0.12% for N_2_ in air at atmospheric pressure, for a one-second measurement interval with a laser power of 150 mW [10]; this corresponds to a 3σ detection limit of 3.6 mbar. Recently, our group demonstrated the use of a capillary system—based on a metal-lined glass capillary of 650mm length—for dynamic, *in situ* process monitoring [11]; and in a system variant suitable for the analysis of tritium-containing, radioactive gases, we were able to detect hydrogen isotopologues with partial pressures of less than 0.5 mbar, for acquisition cycles of 50 × 0.1 s, *i.e.*, providing composition information every 0.1 s by means of a ‘rolling average’ data evaluation procedure [12]. These results demonstrated the potential of the capillary technique for sensitive, real-time process control based on Raman spectroscopy.

Despite the successful use of capillary Raman systems for signal enhancement, one major issue remaining in the development of capillary-based Raman sensors for routine analysis with low detection limits is the occurrence of strong fluorescence in the system. This fluorescence is due to the interaction of the laser beam with optical components made of glass, and the glass capillary tube itself. The sometimes significant increase in background, associated with the (broadband) fluorescence, contributes heavily to the shot noise in the acquired spectra. This then translates into a deteriorating signal-to-background-noise ratio (SNR); as a consequence, the achievable detection limit worsens to values much higher than the gain in signal amplitude would otherwise suggest.

In this work, we have investigated a range of approaches to minimize the influence of fluorescence contributions in a capillary system. It should be pointed out that the generation of fluorescence in glasses is normally less when using longer Raman excitation wavelengths (such as 780 nm or 830 nm); however, on the downside, one is faced with a severe decrease in Raman signal amplitude which follows the approximate ν^4^-law. For this reason, we focus on approaches suitable to minimize fluorescence also for shorter laser excitation wavelength (e.g., 532 nm or lower). We suggest several modifications of the system setup, including re-arrangement and replacement of various optical components and the substitution of the glass capillary by an all-metal light-guide, and discuss the effect of each on both signal and background noise, and hence achievable detection limits. Comparisons with earlier measurements in our group, using pre-optimization capillary systems, are included. These show the new design in a favorable light: improvements in detection limits by about an order of magnitude were demonstrated with a simple prototype setup, with room for further optimization.

## 2. Experimental Section

### 2.1. Capillary Raman Setups

**Figure 1:**
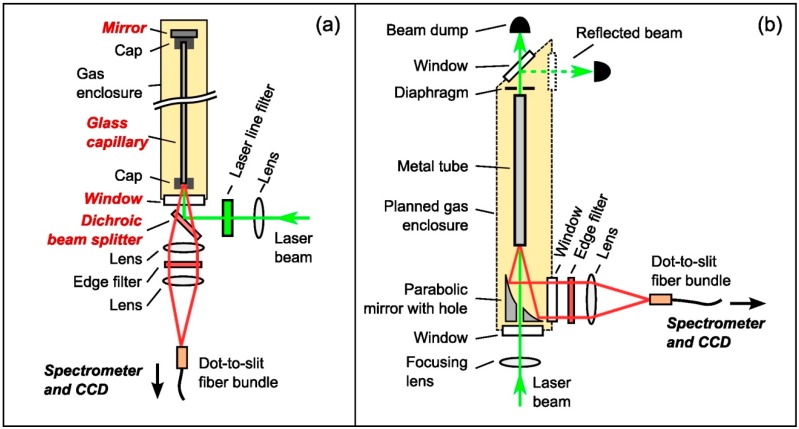
Schematic sketches of the capillary Raman spectroscopy setups. (**a**) The original system used in our laboratory [11,12]; the optical elements annotated in red contribute to the fluorescence background; (**b**) The improved setup resulting from this study; for details see text.

A schematic view of the optical setup of our original capillary Raman setup [11,12] is shown in Figure 1a. The capillary is a 650 mm long, hollow, silver-lined glass fiber (Doko Engineering, Sendai, Japan), with inner diameter of 1 mm and outer diameter of 1.6 mm. The laser used in this work is a 532 nm DPSS Nd: YVO_4_ laser (Laser Quantum, ‘Excel’, providing up to 2 W continuous wave output, Stockport, United Kingdom). The beam is focused into the capillary by a long focal length lens (*f* = 750 mm) to achieve a good coupling of the laser light, with minimal excitation of lossy high-order modes. Fluorescence generated in the focusing lens is rejected by a laser line filter. A dichroic beam splitter (Semrock, Razor Edge LPD01-532RS, Rochester, NY, USA) allows for a spatial separation of laser and Raman light; it reflects the laser into the capillary and couples the back-reflected beam out of the system, while the Raman light can pass through. Two achromatic lenses (*f*_1_ = 75 mm and *f*_2_ = 60 mm, both with diameter *d* = 25.4 mm) collect the Raman light and image it onto a fiber bundle (CeramOptec, Bonn, Germany) consisting of 48 individual fibers of core diameter 100 µm and numerical aperture NA = 0.22, arranged in a custom “dot-to-slit” configuration. The dot-shaped collection end of the bundle has a diameter of just less than 1 mm, similar to the capillary core diameter. The output end is arranged in a slit configuration of around 6 mm in height to fit the vertical sensor dimension of the CCD (charge-coupled device) array detector (Princeton Instruments, PIXIS400B, Trenton, NJ, USA) at the exit of the spectrometer SP500i, with a 600 gr/mm grating (PI Acton, Trenton, NJ, USA). A 532 nm edge filter between the collection lenses (Semrock LP03-532RS-25, Rochester, NY, USA) prevents stray laser light and Rayleigh-scattered light from entering the fiber bundle, to prevent the generation of additional fluorescence light.

The above setup already incorporates several fluorescence reduction measures: caps covering the capillary ends block laser light from directly entering and fluorescence light from exiting the capillary wall face; a thin (thickness = 2 mm) high-purity fused silica cell window was used; and the number of optical components which are exposed to laser radiation, and which are within the collection cone for Raman light, was reduced compared to other direct-focusing capillary setups—such as the one described by Buric *et al.* [10]—by placing the Raman collection lens behind the beam splitter instead of in front. A more detailed description of said fluorescence reduction steps can be found in James *et al.* [11]. However, despite these measures, the fluorescence background of the system was still found to be non-negligible.

**Figure 2:**
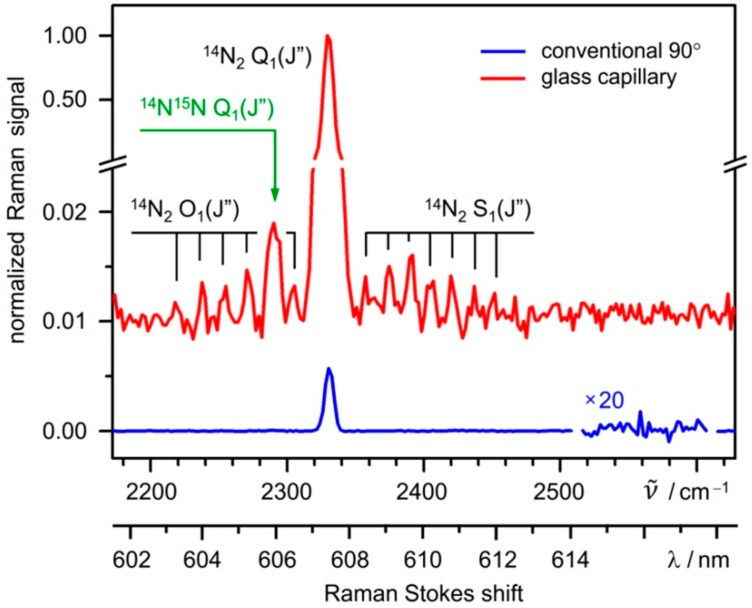
Raman spectra of nitrogen in ambient air, recorded in glass-capillary (*ℓ* = 650 mm) and 90°-cell (*ℓ* = 6 mm) experimental configurations; relevant spectral features are annotated. The spectra were acquired in a direct-comparison measurement using the same laser, spectrometer and detector; five acquisitions of 2 s duration each were averaged for both configurations. The background in both spectra is subtracted, and the capillary measurement data are offset by 0.01 for clarity.

The resulting increase in shot noise becomes apparent in Figure 2, which shows a comparison of (background-subtracted) spectra acquired with the capillary setup and a virtually fluorescence-free conventional 90° Raman setup as described e.g., in [13]. The obvious signal gain (by a factor of ~170) is counteracted by the increase in shot noise (by a factor ~20). Thus, the detection limit, which is associated with the signal-to-background-noise ratio, does not merely reflect the signal gain provided by the capillary system, but is affected by the increased background shot noise as well.

### 2.2. Investigation of Fluorescence-Minimizing Measures

As indicated in Figure 1a, fluorescence contributions in the capillary setup are expected from all glass components which are exposed to laser light: (i) the glass capillary itself; (ii) the dichroic beam splitter; (iii) the window at the capillary entrance; and (iv) the back-reflecting mirror at the exit-end. All of them comprise a coated glass substrate. In the following, measures are described to minimize the fluorescence contributed by these components; the associated results are presented in Section 3.

For the sake of simplicity, the systematic investigations undertaken here were not performed in a closed gas enclosure, as shown in Figure 1, but in an open setup where Raman spectra of ambient air were recorded. Care was taken that all described modifications are suitable for a later integration of the system in a closed gas loop for *in situ* measurements as described in James *et al.* [11]. Note that in these studies a SP2150i (PI Acton) spectrometer was used, instead of the SP500i (PI Acton) in our earlier work.

#### 2.2.1. Alternatives for Laser/Raman Light Separation

**Figure 3:**
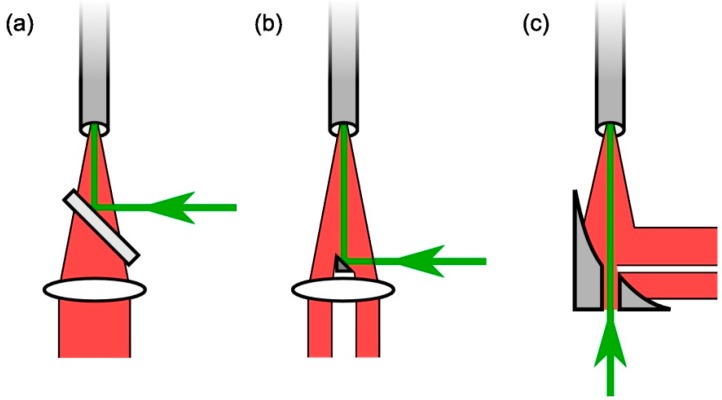
Comparison of laser (green) and Raman (red) light paths when using (**a**) a dichroic beam splitter; (**b**) a pick-off mirror; or (**c**) an off-axis parabolic mirror with central hole for light separation.

The dichroic beam splitter separates laser and Raman light by means of an optical coating, sending the respective wavelength components into different directions. An alternative for this approach would be direct spatial separation of the two light beams, which can be achieved either (i) by a small pick-off mirror; or (ii) by a larger mirror with a central hole, as illustrated in Figure 3. In the former case, the laser beam is reflected into the capillary by the pick-off mirror, while the bulk of the back-scattered Raman light passes around the small mirror and can be collected. In the latter case, the laser beam enters the capillary through a small hole in a mirror which reflects the back-scattered Raman-light in a 90° angle towards a side-arm where the Raman light collection optics are placed.

The drawback of the aforementioned alternatives is that a certain fraction of the Raman light—more specifically the part which is directly back-scattered (scattering angles close to 180°) and which thus suffers the least losses due to reflections in the capillary—is lost for detection: it is either blocked by the laser pick-off mirror, or leaves the system through the laser access hole. For this reason, it is crucial that the dimensions of the pick-off mirror or the hole are not larger than necessary with respect to the laser beam size, and that the distance to the capillary (which is limited by the focal length of the collection lens) is as large as possible. Regardless, however, losses are unavoidable. On the other hand, one gains a significant advantage. In configurations (b) and (c), metal-coated reflective mirrors are used, rather than the dual-wavelength dielectric coating on a glass substrate in configuration (a). While the reflectivity of protected silver (e.g., Thorlabs data sheet for mirror MPD249H-P01, Munich, Germany), with R > 95% over the wavelength range 530–720 nm, is slightly worse than that of the best dielectric coatings (e.g., Semrock data sheet for LPD02-532RS, Rochester, NY, USA) with R > 98% over the same range, transmission through a glass substrate is avoided. Thus, no spurious fluorescence generation in the mirror substrate by even trace-transmission of 532 nm radiation occurs. In our experience, the reduction in fluorescence background far outweighs the slightly lower reflectivity for the metal-coated mirror, so that the overall effect is positive.

In the configuration based on a pick-off mirror (see Figure 3b), a right-angle prism with protected-aluminum coated hypotenuse surface (Knight Optical, PTK0203, Harrietsham, United Kingdom) was used, serving as a 45°-mirror with size 2 × 3 mm It was inserted directly in front of the Raman light collection lens in a custom-made mount, fitting Thorlab’s cage system for conventional 25.4 mm-optics. The prism mirror was attached to the end of a thin metal sheet of thickness *d* = 0.5 mm, reaching from top into center of the mount with the thin side facing the capillary. In this way, only a minimal amount of the Raman light was blocked by the metal holder.

In the configuration based on a mirror-with-hole (see Figure 3c), an off-axis parabolic mirror (Thorlabs, MPD249H-P01: aluminum substrate, diameter *D*_m_ = 50.8 mm, reflective focal length *f* = 101.6 mm) was used, exhibiting an on-axis through-hole of diameter *D*_h_ = 2 mm (custom-dimension). Note that because of the size of this off-the-shelf mirror, the lens imaging the Raman light onto the fiber bundle had to be changed to the same size, to capture the full illuminated area of the mirror; its focal length (*f* = 75 mm) was selected so that the maximum light collection solid angle of the system did not change significantly from our standard 25.4mm-optic setups.

In order to minimize fluorescence contributions from components other than the laser/Raman light-separating component, the cell entrance window and the back-reflecting mirror were not included in the setup used for this investigation; and instead of the glass capillary a silver-metal tube of inner diameter ID = 4.5 mm and polished inner surface, of length *ℓ* = 200 mm, was used. Due to the overly large diameter of the tube the laser beam did not come in contact with the tube walls, and thus no fluorescence from the “capillary” was expected. These measures should allow one to compare only the influence of the beam splitter and its alternatives on background and shot noise. Note that while this large-ID tube guarantees minimal fluorescence generation at the same time it comes at the expense of Raman collection efficiency, due to the severe solid-angle mismatch between the opening of the light-guiding tube and the fiber bundle diameter.

#### 2.2.2. Comparing Metal Capillaries with Metal-Lined Glass Capillaries

Traditionally, capillaries for Raman measurements are usually made of glass with a reflective coating on the inner surface, taking advantage of the intrinsically smooth surface of glass which—together with the metal coating—provides very high reflectivities. Full-metal capillaries/tubes have been used to a much lesser degree, simply because of their rougher inner surface and the difficulties to accomplish a sufficiently good (smooth) internal polish for small inner diameter tubes. Both factors limit the reflectivity within the metal capillary/tube. On the other hand, metal capillaries may seem attractive with respect to spectral shot-noise, since one does not expect major fluorescence contributions from them. Furthermore, they provide potentially better mechanical stability, and are thought to exhibit enhanced longevity of the optical surface in “harsh” environments (e.g., aggressive chemical or radioactive); the durability of dielectric and metal coatings exposed to radioactive tritium is currently being tested in our group.

For these reasons, we investigated metal capillaries as an alternative to the standard glass-based variety. In order to achieve reasonably high reflectivities, so-called “Light Pipes” (manufactured by Epner Technology Inc., Brooklyn, NY, USA) were used. These are nickel tubes lined with a hard gold coating; the coating is produced by galvano-forming, which guarantees that they are highly reflective even for small diameters [14]. It should be noted that the reflectivity of gold (Au) is lower than that of silver (Ag) in the visible range, particularly at the laser wavelength of 532 nm but also for the Raman light (in general in about the range 600–700 nm), although the difference is much less. For the related reflectivities one finds, at normal incidence, R_Ag_ = 98.4% and R_Au_ = 76.1%, and R_Ag_ > 98.8% and R_Au_ > 93.5%, respectively [15]. Despite the lower reflectivity gold coatings are recommended in cases where a protective polymer-overcoat of the capillary’s silver-lining is not possible (such as in tritium systems), to prevent a degradation of the optical properties caused by surface tarnishing.

Three sample capillaries of length *ℓ* = 200 mm each were compared: a glass capillary with inner diameter ID = 1 mm (the same type as used in our previous measurements), and two Light Pipes with ID = 1 mm and ID = 2 mm, respectively. In the Raman setup used for their comparison, the aforementioned mirror-with-hole configuration was implemented instead of the beam splitter, and the front cell-window and back-reflecting mirror were not installed. Thus, exclusively, fluorescence originating from the glass capillary/metal Light Pipe was probed. All capillaries were inserted into a groove, fitting their outer diameter to ensure their straightness. An x-y adjustable 0.8 mm pinhole was placed in front of the capillary to prevent laser light from entering the capillary glass walls directly and fluorescence light from exiting (for details of this measure see James *et al.* [11]). For completeness, a measurement was also taken without any capillary inserted, to ascertain a fluorescence-free base line.

#### 2.2.3. Alteration in Cell Window and Mirror Configurations

In general, in backward-observation configurations with a dichroic beam splitter, as shown in Figure 1a, major fluorescence originates from the window sealing the gas system at the front end of the capillary. This is specifically true, if this window is in close proximity to the capillary orifice, and thus it is close to the focal point of the collection lens.

If a mirror-with-hole is used instead of the beam splitter, as proposed above, another configuration is possible. Assuming that in corrosive environments a full-metal mirror is less prone to damage than a dielectric coating, it can be placed inside the gas system, as shown in Figure 1b above. This has two advantages. Firstly, the laser beam enters the system through one window in front of the mirror-with-hole, which blocks the generated fluorescence almost completely from the line-of-sight of the Raman light collection. Secondly, the Raman light exits the gas system through a second window, which is not exposed to laser light at all.

In order to compare the influence of the gas cell window on the fluorescence signal, depending on its position, once again a silver tube with inner diameter ID = 4.5 mm was used as the capillary and the back-reflecting mirror was removed, as described in Section 2.2.1. The (fused-silica) window was anti-reflection coated for both laser and Raman wavelengths (Thorlabs WG41050-A, Munich, Germany, thickness *d* = 5 mm, coating BBAR = 350–700 nm). This window was placed 11 mm in front of the capillary, corresponding to the distance of the window in the original setup (as in Figure 1a), or it was placed directly in front of the mirror-with-hole (as in Figure 1b).

The setup in Figure 1a which was used in our earlier work has the bonus of a back-reflector, effectively doubling the interaction length with the laser radiation. In the revised setup (Figure 1b) that internal reflector was removed and the capillary cell was closed with an exit window. This allows for easier alignment of the laser beam traversing the light-guide; an optional, external laser back-reflector can be added if required. On the downside, any back-reflection from components at the rear end of the system (e.g., the directly-coupled mirror or a cell window with external mirror) can induce additional fluorescence, and therefore raise background levels and shot noise. In particular, one should be able to minimize contributions from the window (i) by tilting it with respect to the laser beam axis, so that laser beam reflections are not coupled back into the capillary; and (ii) by adding a diaphragm between the rear window and the capillary, to minimize the capture of fluorescence generated within it. In order to test the light return from optical components (windows) at the exit end of the capillary, a thin, uncoated fused-silica window (Thorlabs WG41010, thickness d = 1 mm) was placed at a distance of 30 mm behind the capillary exit. Starting at perpendicular laser beam incidence, it was tilted in steps of 5° with respect to the laser beam axis; in addition, a measurement without the rear window was made to provide a “base-line” value, free of any back scattering.

### 2.3. Data Acquisition and Analysis

For the comparison measurements addressing the above issues, Raman spectra of air were acquired for each configuration, using a fixed set of operating parameters. Unless stated otherwise, the following settings were used: (i) the 532 nm laser power was kept at 1 W; (ii) the CCD detector was cooled to −73 °C to reduce thermal noise; (iii) the 400 vertical CCD pixels were combined to 25 bins of 16 pixels each to reduce the read-out noise, but still allow for astigmatism-correction in the case that high-spectral resolution was desired; (iv) 50 acquisitions of 1s each were averaged for noise reduction; and (v) to quantify thermal and read-out noise, dark measurements were performed during each measurement series, with the laser turned off.

The spectroscopic data for air at atmospheric pressure were acquired and processed using our integrated acquisition and analysis routine LARASoft (Karlsruhe, Germany) [4], which includes cosmic-ray and background removal routines, as well as peak evaluation. After the removal of the cosmic-ray spikes and the background pedestal, two quantities were extracted, namely the signal-peak amplitude and the background noise, to be used for the prediction of quantitative analysis capabilities. The signal amplitude *I*_signal_ was extracted by taking the maximum intensity of the (rotationally unresolved) Q_1_-branch of N_2_, with the background subtracted. The background noise *I*_noise_ was determined by taking the standard deviation of a flat part of the spectrum close to the N_2_ (Q_1_) Raman peak, but beyond its O_1_- or S_1_-branches.

At this point, it is worthwhile to add a few short remarks on signal fluctuations, or noise. The overall fluctuations include the intrinsic signal shot-noise, $\sigma_{\text{S}}$, and extrinsic noise contributions, comprising external background noise from e.g., material fluorescence, room light, or other sources (all governed themselves by shot-noise), $\sigma_{\text{B}}$; and detector noise in the form of thermal, $\sigma_{\text{T}}$, and read-out noise, $\sigma_{\text{R}}$. Assuming that all noise contributions follow stochastic statistical behavior, the total noise distribution can be described via the convolution of the individual contributions, *i.e.*

(1) $$\sigma_{\text{total}}\;={\left[\sigma_{\text{S}}^{2}+\underset{background\;noise}{\underset{︸}{\left(\sigma_{\text{B}}^{2}+\sigma_{\text{T}}^{2}+\sigma_{\text{R}}^{2}\right)}}\right]}^{1/2}$$

It is the background noise alone—*i.e.*, $\sigma_{\text{b}}\;={\left[\sigma_{\text{B}}^{2}+\sigma_{\text{T}}^{2}+\sigma_{\text{R}}^{2}\right]}^{1/2}$ (with any signal absent)—which is commonly used in analytical procedures to determine the limits of detection (LOD) and quantification (LOQ). Since it was the goal of this study to ascertain whether our improvement measures yielded indeed improved LODs, throughout this work we have utilize the related ratio of signal amplitude to background noise fluctuation (*i.e.*, signal-to-background-noise ratio ≡ SNR), as recommended by IUPAC (International Union of Pure and Applied Chemistry) in their guidelines [16], and expanded on by Voigtman [17]. Based on these SNR values, the 3σ-LOD for N_2_ was calculated for each instrumental test configuration, *i.e.* relating it to the partial pressure *p* which corresponds to a signal-to-background-noise ratio with value SNR = 3:

(2) $$\text{LOD}=\left(3p/\text{SNR}\right)=3p\cdot \left(I_{\text{noise}}/I_{\text{signal}}\right)$$

Note that the calculation of the signal uncertainty, in our analysis, takes into account the shot-noise, the CCD thermal and read-out noise, and the uncertainty in laser power. For the latter, an upper limit of 1% was estimated, which dominated the overall signal uncertainty. The uncertainty of the background noise was taken as the standard deviation of the mean of a signal-free part of the spectrum.

## 3. Results and Discussion

### 3.1. Comparison of Dichroic Beam Splitter, Pick-Off Mirror and Mirror-With-Hole

The results from the comparison measurement between the beam splitter and its alternatives are listed in Table 1. The tabulated data reveal the following. For the pick-off mirror, a considerable fraction of the total signal was lost, due to Raman light being blocked by the mirror itself and its mount. For the mirror-with-hole, the signal reached levels which were comparable to those achieved using the dichroic beam splitter.

**Table 1:** Comparison of the influence of fluorescence from a dichroic beam splitter, and its replacement alternatives, on the signal-to-background-noise ratio (SNR) of capillary Raman measurements.

	*I*_signal_ (10^3^ counts)	*I*_noise_ (counts)	SNR (10^3^)
Beam splitter	98.72 ± 0.99	5.8 ± 0.7	17.0 ± 2.2
Pick-off mirror	70.69 ± 0.71	7.2 ± 0.9	9.8 ± 1.2
Mirror-with-hole	98.76 ± 0.99	2.8 ± 0.4	35.3 ± 4.5
Dark measurement	---	2.6 ± 0.3	---

Interestingly, when a pick-off mirror was used, the noise level increased, indicating that the fluorescence had become worse. This is due to the fact that laser stray light could easily interact with both the glass substrate of the mirror and the collection lens right behind the mirror. In contrast, for the metal-based mirror-with-hole the noise level diminished practically to the level of a dark spectrum, *i.e.*, the thermal and read-out noise from the detector. These findings clearly demonstrate that fluorescence contributions from the wavelength-separating element can be eliminated nearly completely if a full-metal component is used instead of glass-substrate optics, and that the achieved SNR has about doubled. Thus, the use of a mirror-with-hole instead of a standard dichroic beam splitter is strongly recommended in capillary Raman setups.

### 3.2. Comparison of the Performance of Metal-Lined Glass and Full-Metal Capillaries

The results of the comparison between the silver-lined glass capillary and the two Light Pipes are shown in Table 2. As in previous experiments, expectedly, the silver-lined glass capillary exhibits a significant Raman signal enhancement in comparison to the “no-capillary” configuration. While the enhancement is still dramatic for the gold-lined metal pipes, it is significantly less than that for the glass capillary. One very likely reason for this is the higher reflectivity of silver (R_532_ ≈ 0.96–0.98) compared to gold (R_532_ ≈ 0.75) at the laser wavelength λ = 532nm. For example, the enhancement difference between the glass and metal capillaries with the same ID = 1 mm is very much in line with the difference in reflectivities. In addition, the higher surface smoothness of glass compared to the inner surface of a metal tube may contribute to a lowering of the guided signal.

**Table 2:** Performance of metal-lined glass capillary and metal Light Pipes with different inner diameters ID, in comparison with a 180° Raman measurement without light-guide.

	ID (mm)	*I*_signal_ (10^3^ counts)	*I*_noise_ (counts)	SNR (10^3^)
Glass capillary	1	317.65 ± 3.18	26.2 ± 3.4	12.1 ± 1.6
Light Pipe	1	235.86 ± 2.36	10.9 ± 1.4	21.6 ± 2.8
Light Pipe	2	178.15 ± 1.78	4.7 ± 0.6	37.9 ± 4.9
No capillary	---	33.41 ± 0.34	2.8 ± 0.4	11.9 ± 1.6
Dark measurement	---	---	2.7 ± 0.3	---

If the inner diameter of the Light Pipe is increased, the signal drops even further; this is because the Raman light cannot be completely imaged onto/coupled into our light collection fiber bundle of *d*_fb_ = 1 mm.

However, it is the (fluorescence) noise reduction which gives the metal Light Pipes the advantage over a metal-lined glass capillary. The statistical background noise reduces by more than a factor of two when replacing the ID = 1 mm glass capillary with an ID = 1 mm Light Pipe, and diminishes by a further factor of about two when changing to a Light Pipe with ID = 2 mm. The reduction in the latter is linked to the lesser interaction of the laser beam with the inner tube surface. The signal loss of about 45% is more than compensated by a noise reduction of >80%. As a consequence, this leads to an overall improvement in SNR by more than a factor of three.

We would like to finish this section with a few general remarks on the ID-parameter for light-guides. Recall that the measurements described earlier in Section 3.1 utilized a solid-silver metal tube with ID = 4.5 mm. This was done in order to completely eliminate the fluorescence background from the capillary itself, but at a price: the signal amplitude was significantly reduced. Comparing the data in Table 1 and Table 2, one has to come to the conclusion that, with further increases from said tube diameter, one will at some stage reach the limit of the “no capillary” scenario.

Optimally, the capillary/Light Pipe diameter should be chosen such that it is sufficiently large to prevent background (fluorescence) generation from the device itself, while still providing reasonable laser and Raman light guiding to achieve signal enhancement. For standard laser systems, this implies larger diameters than have been used in capillary Raman systems reported in the literature. A reduction of the laser beam diameter is in principle also possible, but normally this goes along with a larger beam divergence, which as a consequence leads again to laser interaction with the capillary wall, at least for long capillaries.

Note that despite the signal reduction associated with the poorer imaging properties, a tube diameter of few millimeter could still be advantageous for a range of applications relying on *in situ* measurements of flowing gases. Larger inner tube diameters allow for a higher gas throughput, and help to prevent differential pumping and gas-separation effects, which might otherwise influence the local composition of the very gas which is to be analyzed.

Finally, another advantage of a large-core light-guide is—despite the reduced Raman light collection efficiency—the possibility to significantly increase the laser power, without damaging the capillary/Light Pipe coating. With the fluorescence background nearly completely eliminated, a simple increase in laser power will lead to a higher Raman signal, but not a higher noise level; hence one can achieve the same relative increase in SNR, and thus lower the detection limit by about the same factor.

### 3.3. Comparison of Raman Cell-Window Configurations

Finally, the influence of the front and rear windows on the fluorescence background was investigated; the results are summarized in Table 3.

The comparison between the old front-window position, *i.e.*, directly in front of the capillary, and the revised position, where the window is placed before the mirror-with-hole, is shown in the top half of Table 3. While the signal amplitude is comparable for the two configurations, the noise differs considerably: for the new configuration, it is now at the level of the dark measurement, yielding a vast improvement, almost by a full order-of-magnitude. Thus, the suggested and tested window repositioning is an important step towards a fluorescence-free capillary Raman system.

For the optional rear window (note that this was not implemented in our original capillary setup in which an internal high-reflector was mounted inside the cell instead), it was found that the fluorescence due to back-reflected laser light could be successfully decreased by tilting the window. The results for the rear window tilt measurements are shown in the bottom half of Table 3. Here, the measurement at a tilting angle of 45° was chosen as an example, since the results for all tilting angles were very similar, most of them within the uncertainty of the measurement. Note that the signal is slightly higher for the perpendicular window; this is due to the Raman contribution of the reflected laser beam instigating an additional amount of Raman light. On the other hand, the fluorescence background from this line-of-sight orientation is increased. Overall, it was evident from the measurements that the noise level can be decreased almost to the level of a dark measurement by avoiding these back-reflections; this in turn leads to higher SNR.

**Table 3:** Influence of the front and rear cell windows (position and orientation) on the fluorescence background.

	*I*_signal_ (10^3^ counts)	*I*_noise_ (counts)	SNR (10^3^)
Front cell window			
In front of capillary	97.82 ± 0.98	27.9 ± 3.6	3.5 ± 0.5
Before mirror-with-hole	93.32 ± 0.93	2.8 ± 0.4	33.3 ± 4.3
Dark measurement	---	2.7 ± 0.3	---
Rear cell window			
Perpendicular laser incidence	99.72 ± 1.00	8.7 ± 1.1	11.5 ± 1.5
Window tilted by 45° to beam	94.41 ± 0.94	3.5 ± 0.5	27.0 ± 3.5
No window	95.23 ± 0.95	2.8 ± 0.4	34.0 ± 4.4
Dark measurement	---	3.1 ± 0.4	---

It should be noted that the small amount of remaining background is fluorescence light originating from components behind the capillary, such as the window itself or a laser beam dump. Parts of the emitted light couples into the capillary and is thus detected. It is therefore advisable to place such components as far from the capillary end as the system design allows. If that is not possible, we demonstrated that by adding a diaphragm or pinhole directly behind the capillary end, the fluorescence light can be limited as well: the suitable choice pinhole diameter just allows the laser beam to exit, but blocks most background light originating from beyond it from being directed back through the capillary.

We achieved full elimination of detected background light when the distance of the tilted window from the capillary end was at least 60 mm, and a diaphragm was used in addition. It goes without saying that any anti-reflection coating for the tilted rear window should be suitable for non-perpendicular incidence.

### 3.4. Evaluating the Overall Sensitivity Improvement and Gauging the Suitability of the Revised Setup for Real-Time Measurements

To evaluate the overall effect of the suggested alterations on the detection limit of the capillary Raman system, measurements using the original setup (as described in Section 2.1) were compared with measurements using the improved prototype, with all fluorescence-reducing measures implemented. The two configurations are shown in Figure 1 above; note that the Light Pipe with inner diameter ID = 2 mm and length *ℓ* = 200 mm was used as metal capillary in the improved setup.

The results of these comparison measurements are collated in Table 4. It should be noted that the spectrometer used in the original setup had a lower light throughput of only around 38% of the one used in the measurements with the improved setup. The spectrometer throughput affects both the signal and noise components since less light is collected. Accordingly, for a sensible comparison, the data have to be normalized (scaled) to each other. The scaled data for the original system measurement is tabulated in the second row of Table 4. Note that the scaling factor to account for the higher light-throughput spectrometer in the improved system was 2.64 for the signal and √2.64 for the noise (assuming Poisson-distributed shot-noise, being proportional to the square-root of the fluorescence background [18]).

In addition, in deviation from the commonly adopted measurement parameters used for all comparison measurements discussed in the previous sections (listed in Section 2.3), some parameters were selected to gauge the system-suitability for real-time measurements, in addition to the sensitivity comparison. For this, the number of on-chip pixel bins was reduced from 25 to just five, thus increasing the readout speed of the CCD detector, and the spectrum accumulation time was lowered from 1 s to 0.37 s (which approximately matches the minimum time required for combined data read-out and evaluation, in parallel to the next signal accumulation).

**Table 4:** Comparison of the original system and the new prototype with all fluorescence-reducing improvements implemented.

	*I*_signal_ (10^3^ counts)	*I*_noise_ (counts)	SNR (10^3^)	LOD (mbar)
Original system	112.82 ± 1.13	39.0 ± 5.0	2.9 ± 0.4	0.81 ± 0.10
Original system (scaled)	297.83 ± 2.98	63.4 ± 8.1	4.7 ± 0.6	0.50 ± 0.06
Improved prototype	59.35 ± 0.60	1.8 ± 0.2	33.0 ± 4.1	0.07 ± 0.01
Dark measurement	---	1.6 ± 0.2	---	

As expected, the signal obtained with the original setup exceeded the one from the new prototype setup. This is partly due to the reasons discussed above, *i.e.*, the lower reflectivity of the gold-lined Light Pipes for the laser wavelength, as well as a reduced coupling of Raman light into the fiber bundle leading to the spectrometer. Apart from this, the laser beam passes the capillary twice in the original setup (a back-reflecting mirror is incorporated, see Figure 1a), and the length of the capillary is 650 mm instead of just 200 mm for the Light Pipe, so that the interaction region in the original setup is much larger than that of the current prototype. It should be noted that the short (for reasons of cost) Light Pipe units were originally acquired to test their suitability for capillary Raman spectroscopy. Of course, in a “final” setup, much longer units could be incorporated, so that a further increase in signal and hence detection sensitivity is expected.

Despite the shorter light-guide length in our prototype setup, the lower signal—in comparison to the 650 mm metal-lined glass capillary—is more than compensated for by the considerable noise reduction by more than one order-of-magnitude. The data show that the improvements presented here allow one to realize a fully-fledged capillary system with noise levels approaching that of the base detector noise, practically free of shot-noise contributions due to fluorescence light. Overall, we demonstrated that the limit of detection could be improved by more than one order-of-magnitude (by a factor of ~7 if the higher throughput spectrometer is taken into account) compared to the previous setup, even with a shorter capillary.

Finally, using the common rolling average approach [19], detection limits of <0.1 mbar in less than 0.5 s of feedback time can be reached. Even without the rolling average, if just a single spectrum of 0.37 s acquisition time is evaluated, we reach LOD = 0.51 mbar for N_2_. This shows that with our revised capillary Raman system sub-mbar detection limits can easily be achieved in sub-second measurement times. Considering the prototype character of the setup and the possibilities to further increase the signal, by optimizing diameter and length of the capillary, even better detection limits are expected. Our results suggest that the capillary system presented here constitutes a feasible candidate for *in situ* real-time measurements, coupled with high sensitivity, e.g., for rapid process control and trace gas analysis.

## 4. Conclusions

In this work, we have systematically worked towards a minimization of fluorescence in a capillary/light-guide Raman system for high-sensitivity gas analysis. We have suggested and experimentally tested different fluorescence reducing measures, such as the rearrangement of optical components to reduce the production and detection of fluorescence light, and the replacement of glass components by suitable metal alternatives. Based on these investigations, we were able to set up a prototype of an improved capillary Raman system in which fluorescence background—common to standard glass capillary systems—was practically eliminated, while at the same time reasonably high Raman signals could be obtained. The sum of all these improvements led to a limit of detection (LOD) which was a factor of about seven better than that achievable with our previous system based on a standard glass capillary setup, although our prototype all-metal Light Pipe was substantially shorter than our normal metal-lined glass capillary.

With the improved setup, LODs well below 1 mbar could be achieved, for sub-second acquisition times. It should be noted that the measurements presented here were performed with a prototype setup aiming primarily for the demonstration of the fluorescence minimization, and initially not for signal maximization. Clearly, further enhancement of the signal is possible by optimizing the length and diameter of the Light Pipe, and by using higher laser powers. Thus, even better detection limits can be expected.

The fluorescence-minimized Light Pipe capillary Raman system presented here combines all advantages of Raman spectroscopy, such as simultaneous multi-species detection, with a robust optical setup suitable for integration into e.g., gas processing loops. Our results (obtained with a far from perfect prototype setup) suggest that capillary Raman spectroscopy constitutes a promising candidate for *in situ* real-time measurements with high sensitivity, e.g., for rapid process control and trace gas analysis.

## References

[1] Marteau P., Zanier-Szydlowski N., Aoufi A., Hotier G., Cansell F. (1995). Remote Raman spectroscopy for process control. Vib. Spectrosc..

[2] Csontos I., Pataki H., Farkas A., Bata H., Vajna B., Nagy Z.K., Keglevich G., Marosi G.J. (2015). Feedback control of oximation reaction by inline Raman spectroscopy. Org. Process Res. Dev..

[3] Atkins P.W., de Paula J. (2006). Physical Chemistry.

[4] Schlösser M., Bornschein B., Fischer S., James T.M., Kassel F., Rupp S., Sturm M., Telle H.H. (2015). Raman spectroscopy at the Tritium Laboratory Karlsruhe. Fusion Sci. Technol..

[5] Taylor D.J., Glugla M., Penzhorn R.D. (2001). Enhanced Raman sensitivity using an actively stabilized external resonator. Rev. Sci. Instrum..

[6] Ghaziaskar H.S., Lai E.P.C. (1992). Stimulated Raman scattering in analytical spectroscopy. Appl. Spectrosc. Rev..

[7] Stiles P.L., Dieringer J.A., Shah N.C., Van Duyne R.P. (2008). Surface-Enhanced Raman Spectroscopy. Annu. Rev. Anal. Chem..

[8] Carlsen W.F., Simons T.D., Pittaro R.J., Hopkins G.W., Gray D.F. (1996). System for collecting weakly scattered electromagnetic radiation. U.S. Patent.

[9] Pearman W.F., Carter J.C., Angel S.M., Chan J.W. (2008). Quantitative measurements of CO_2_ and CH_4_ using a multipass Raman capillary cell. Appl. Opt..

[10] Buric M.P., Mullen J., Woodruff S.D., Chorpening B. Design and industrial testing of ultra-fast multi-gas Raman spectrometer. Proceedings of the SPIE Defense, Security, and Sensing.

[11] James T.M., Rupp S., Telle H. (2015). Trace gas and dynamic process monitoring by Raman spectroscopy in metal-coated hollow glass fibres. Anal. Methods.

[12] Rupp S., James T.M., Telle H.H., Schlösser M., Bornschein B. (2015). Enhanced sensitivity of Raman spectroscopy for Tritium gas analysis using a metal-lined hollow glass fiber. Fusion Sci. Technol..

[13] James T.M., Schlösser M., Fischer S., Sturm M., Bornschein B., Lewis R.J., Telle H.H. (2013). Accurate depolarization ratio measurements for all diatomic hydrogen isotopologues. J. Raman Spectrosc..

[14] The Midas touch for laser cavities and sensing applications. http://www.laserfocusworld.com/articles/print/volume-50/issue-04/world-news/photonic-materials-epner-gold-the-midas-touch-for-laser-cavities-and-sensing-applications.html.

[15] Babar S., Weaver J.H. (2015). Optical constants of Cu, Ag, and Au revisited. Appl. Opt..

[16] Fassel V.A. (1976). International Union of Pure and Applied Chemistry (IUPAC), Analytical Chemistry Division, Commission on Spectrochemical and Other Optical Procedures for Analysis: Nomenclature, symbols, units and their usage in spectrochemical analysis—II. Data interpretation. Pure Appl. Chem..

[17] Voigtman E. (2008). Limits of detection and decision. Spectrochim. Acta B.

[18] Demtröder W. (2008). Laser Spectroscopy, Vol. 2: Experimental Techniques.

[19] Brandt S. (2014). Statistical and Computational Methods for Scientists and Engineers.

